# Bidirectional Associations of Recreational Sedentary Screen Time and 24-hour Behaviors: a dynamic cross-sectional multilevel model analysis

**DOI:** 10.21203/rs.3.rs-6803906/v1

**Published:** 2025-06-23

**Authors:** Kristina Hasanaj, Krista S. Leonard, Dorothy D. Sears, Fang Yu, Megan E. Petrov, Sarah K. Keadle, Matthew P. Buman

**Affiliations:** Northwestern University Feinberg School of Medicine; Arizona State University; Arizona State University; Arizona State University; Arizona State University; California Polytechnic State University; Arizona State University

**Keywords:** screen time, television watching, TV viewing, sedentary behavior, physical activity, sleep

## Abstract

**Background:**

Recreational sedentary screen time (rSST) is the most prevalent discretionary sedentary behavior and is strongly linked to poor health outcomes, but how time spent in rSST relates to other 24-hr behaviors is not well understood. The purpose of this study was to examine within and between day associations between rSST and other 24-hr behaviors– non-rSST or other sedentary time (other-SED), standing (STAND), light-to-moderate-vigorous physical activity (LPA), moderate-to-vigorous physical activity (MVPA), and total sleep (SLEEP).

**Methods:**

Baseline data from participants randomized in the StandUPTV study, an intervention that aimed to reduce rSST in adults, were included. All 24-hour behaviors were assessed continuously for 7-days. The activPAL was used to assess rSST, other-SED, STAND, LPA, and MPVA; SLEEP was assessed using a GENEactiv accelerometer. rSST was collected using WiFi plugs to capture TV time and tablet app usage. A multilevel modelling approach was used to assess between(across persons)- and within(across days)-person level associations bidirectionally between rSST (total, daytime, evening) and 24-hour behaviors, and adjusted for age, sex, chronotype, education level, and week vs. weekend day. The results were scaled hourly for interpretation.

**Results:**

On average, 8.0 ± 1.6 days of continuous daily 24-hour behavior data were included from 94 participants (aged 23–64 years [*M* ± *SD*: 42.3 ± 11.5]; 82% female; 78% White; BMI 20.5–67.5 kg/m^2^ [*M* ± *SD*: 29.8 ± 7.8]). Greater total rSST was significantly associated with less other-SED (between-person b=−45.0, SE = 4.4, p < 0.01; within-person b=−44.5, SE = 2.0, p < 0.01). Similar results were observed when examining both daytime and evening rSST with other-SED. Negative associations were also observed between other-SED, STAND, LPA, and MVPA with rSST variables. No significant associations were observed between rSST variables and SLEEP.

**Conclusions:**

This is the first known analysis of the bidirectional relationship between rSST and 24-hour behaviors. More rSST was associated with less other-SED, suggesting rSST may displace rather than contribute to more cumulative sedentary time. These findings suggest that contexts of sedentary behavior should be considered as distinct behavioral targets in intervention development. Future interventions that target rSST reduction should also include strategies that are designed to reduce total sedentary time.

## BACKGROUND

A typical 24-hour day can be categorized into different types and intensities of physical behaviors, including light-intensity physical activity (LPA) or moderate-to-vigorous physical activity (MVPA), standing, sedentary time, and sleep, that differ in their associations with health outcomes.^1,2^ For example, high levels of sedentary time, low levels of MVPA, and short sleep duration are associated with an increased risk of all-cause mortality, cardiovascular disease, metabolic syndrome, type 2 diabetes (T2D), and certain cancers.^1–10^ Replacing sedentary time with more physical activity or compensating for excessive sitting with high MVPA is associated with significant decreases in all-cause mortality in less active adults.^11,12^

Since total time is constrained, increased sedentary time is associated with less time spent in other 24-hour behaviors, such as total physical activity, LPA, and sleep duration.^13^ Indeed, observational evidence suggests that the reallocation of time from sedentary to other physical activities is associated with better health outcomes, including reduced risk for reduced all-cause mortality,^14^ and cardiovascular disease and cancer mortality.^15^ However, these studies typically focus on average displacement effects based on weekly data. The relationship between time-use behaviors is thought to be dynamic and reciprocal; for example, longer sleep duration at night may lead to less sedentary time and MVPA the next day.^16^ These associations are inconsistent and may depend on the age and sex of the participants, the timing of assessment (weekday vs. weekend), type of assessment (reported vs. device-based), and chronotype (i.e., individualized preference of the timing for sleep and waking activity across 24-hour).^17–23^

Additionally, while previous research has examined total sedentary time in relation to other 24-hour behaviors,^17–23^ understanding the predominant discretionary sedentary behavior-recreational sedentary screen time-impacts on time spent in other 24-hour behaviors is not well understood. Recreational sedentary screen time (rSST) is defined as discretionary (i.e., not for work or educational purposes) time spent watching screens, such as television (TV), computers, smartphones, tablets, or inactive video games. rSST is the most prevalent discretionary sedentary behavior outside of work and sleep, consuming over 4.5 hours/day among American adults.^24–26^ rSST is also associated with greater health risks than work or other contexts of sedentary time (e.g., work, transport).^12,14,15,27–33^ Notably, engaging in MVPA at 2–3 times greater than the current physical activity guidelines recommendations^34,35^ (~ 60–75 minutes/day of walking) lowered but did not fully attenuate the increased mortality risk from 5 + h/day of rSST.^14^ Given the increasing prevalence of rSST and its robust association with adverse health outcomes, understanding the influence of this specific and contemporary context of sedentary time on 24-hour behaviors is critical for developing effective domain-specific interventions to reduce sedentary behavior and increase physical activity.

The relationship between time spent in rSST and 24-hour behaviors between- and within-participants is not well understood. Understanding these relationships is necessary to discern if specific behavior context considerations are needed for successful intervention development.^2^ To our knowledge, no studies have examined relationships between rSST and 24-hour behaviors using objective measures of both screen time and physical behavior. The purpose of this study was to investigate the bidirectional associations between rSST and 24-hour behaviors among individuals who reported high levels of screen time using continuous data from device-based measures.

## METHODS

### Study Design and Procedure

This secondary analysis utilized baseline data from the StandUPTV randomized trial. The protocol and main outcomes are described elsewhere.^36^ The StandUPTV study was a full factorial trial that aimed to develop an optimized intervention to reduce rSST in adults by > 60 minutes/day at 16 weeks. Participants were recruited through social media, research matches, newsletters, and online advertisements. After providing informed consent, participants were mailed a technology kit that included smart home devices (i.e., Raspberry Pi network device and WeMo smart plugs for each primary TV used), a pre-loaded tablet with regularly used applications (app), and a Fitbit Charge 4 (Fitbit LLC, San Francisco, CA), which collectively allowed for continuous real-time sedentary screen time tracking. A staff-assisted setup was provided to enable and confirm proper technology set up for rSST measurement. Sociodemographic and other REDCap-administered survey assessments, clinical measures, and device-based measurements (activPAL, GENEActiv) were completed at baseline, 8-, and 16-weeks. This study only used data collected at baseline.

The study protocol was reviewed and approved by the Arizona State University Institutional Review Board (IRB #00012109). All participants received a full explanation of study procedures and potential risks and provided informed consent prior to any data collection. This research was conducted in accordance with the Declaration of Helsinki and relevant U.S. federal regulations governing human subjects research.

#### Participants.

Eligible participants were healthy adults aged 23–64 years.^36^ Inclusion criteria included self-reporting > 3 hours/day of rSST, physical activity during leisure^37^ and occupational time^38^ categorized as below moderate activity, use of an Apple (iOS6+) or Android (2.3+) smartphone or tablet, access to home broadband internet or an unlimited data plan, ability to read and understand English, and willingness to be randomized to any StandUPTV study conditions.

### Measures

#### Recreational Sedentary Screen Time (rSST).

*This was assessed using* an integrated measure of minute-level posture and recreational screen time.

- recreational screen time : recreational screen time was defined as a discretionary activity with screens for enjoyment that included socialization and communicating (e.g., Instagram, Facebook), TV viewing, video games, or leisure computer use. Screen time activities for education (e.g., school, training) and work-related activities were not included as rSST time. Three screen time categories were determined: TV/video, social media, and video games. Screen time was directly assessed using WiFi plugs to monitor home television power state (WeMo Insight Smart Plug, Belkin International, Inc; Playa Vista, CA) and tablet app usage (Samsung Galaxy, Samsung). Smartphone usage was captured via summary metrics from the participant’s smartphone device. Participants could also log or adjust bouts of screen time that occurred outside the home or on alternative devices through the StandUPTV app. Screen time bouts viewed by another household member could also be rejected on the app.
- sedentary time: participants were asked to wear the activPAL3c micro accelerometer (PAL Technologies Ltd, Glasgow, Scotland) continuously during assessment periods to capture sedentary posture (i.e., any waking behavior in a seated or reclining posture at low energy expenditure)^39^. The device attachment procedures were based on several large-scale intervention studies.^15,28,40^ The devices were waterproofed using a medical-grade adhesive covering and attached to the midline of the thigh with breathable, hypoallergenic tape to allow for continuous 24-hour wear for 7 consecutive days at a minimum without removal for bathing or other water-based activities.^36^

The screen time and activPAL data were merged at the minute level to determine rSST. An observation was labeled rSST if both conditions were true: 1) behavior was identified as sedentary by the activPAL, and 2) recreational screen time was identified by either the TV or tablet. Additional rSST variables include daily evening rSST minutes, which were the total daily minutes of rSST that occurred between 5pm and sleep time. Daily daytime rSST minutes were calculated by subtracting evening rSST from total rSST minutes. rSST focused on waking hours only (non-sleep), which was determined using a REDCap-based daily log to identify sleep and wake times.^36,41^

#### non-rSST Sedentary Time, Standing, and Physical Activity.

activPAL3c data were used to further categorize waking behavior (software version 8.12, CREA algorithm). Any sedentary minute without recreational screen time was categorized as non-rSST sedentary time (other-SED). Active time was categorized into standing (STAND), LPA (stepping < 100 steps/minute), and MVPA, with a cadence at ≥ 100 steps/minute.^42^ Cycling was summarized with MVPA. Wear periods were excluded if continuous sitting or standing behavior was > 6 hours (considered non-wear), days had < 10 hours of valid waking wear time, and participants had < 4 valid days.

#### Sleep.

The wrist-worn GENEActiv (initialized to collect at 40 Hz) accelerometer (ActivInsights, Kimbolton, UK) was worn continuously (24 hours/day) for a minimum of 7 consecutive days. The GENEActiv device is validated for sleep,^43–46^ and the daily log completed by participants was used to differentiate sleep and wake periods.^41^ An open-source and validated algorithm (GGIR package, R-software)^46^ was used to process device data and daily log information to provide sleep measures that include sleep duration, efficiency, latency, and wakefulness after sleep onset.^47–50^ Sleep duration is defined as the minutes accumulated within the main sleep window. GGIR signal processing includes autocalibration using local gravity as a reference,^48^ detections of sustained abnormal high values, non-wear detection, and the average magnitude of dynamic acceleration calculation, corrected for average gravity over 5s epochs and reported in milligravitational units (mg). Data files were excluded if the post-calibration error was > 0.01 g (10 mg), fewer than 1 day of valid wear,^47,51^ or wear data were not present for each 15-minute period of the 24-hour cycle. Non-wear detection is described elsewhere.^48^ The default non-wear setting was used, including invalid data imputed by the average at similar time points on different days during the assessment week.^48^ Sleep duration was calculated using automated sleep detection (HDCZA sleep detection algorithm)^50^ for missing log wake/sleep times. Nights with an average sleep duration of ≥ 200 and ≤ 840 minutes were included. Available nightly sleep duration (SLEEP) data were merged with corresponding daily behaviors.

#### Daily 24-hour Behavior Summary.

The data detailed above were joined to create a daily 24-hour behavior summary that included daily total rSST, daytime rSST, evening rSST, other-SED, STAND, LPA, MVPA, and SLEEP (subsequent night) variables. A shifted dataset was also created so that 24-hour behaviors were merged with the previous night’s sleep. Data from participants with ≥ 4 days of valid wear (≥ 10 hours of waking wear from activPAL and screen time data) were included in the analysis.

#### Demographic variables.

Demographic variables were assessed to understand sample characteristics and potential confounders, including age, race/ethnicity, sex, marital status, living arrangements, education, work status, occupation, and household income.

#### Chronotype.

The Morningness-Eveningness Questionnaire (MEQ) was used to assess chronotype.^52,53^ This 19-item validated questionnaire assesses sleep habits, fatigue, and individual differences to understand the degree to which one is more active and alert at certain times of the day. The responses determine preferences in sleep and waking times and the subjective ‘optimal’ time when individuals feel at their best. Based on scores, individuals are classified as either evening type (E-type; ≤52), intermediate or day type (D-type; 53–64), or morning type (M-type; ≥65).^54^

#### Body Mass Index.

Self-reported height and body weight were used to calculate body mass index (BMI). Height was self-reported in inches. Body weight in pounds was collected using the Tanita BF-679 (Tokyo, Japan) scale provided to them. All participants were asked to collect two measurements of body weight and take a photo of the scale display for each measurement and then upload the photos to the REDCap Clinical Measures Log. The research staff reviewed the submitted photos and entered the values, and the average weight was determined from the mean of the weight values provided. Average weight and self-reported height were used to calculate BMI (kg/m^2^).

### Data Analysis

Participants with ≥ 4 days of ≥ 10 hours of valid waking wear time from the activPAL and rSST data, and ≥ 1 valid nightly sleep data were included in this analysis. The bidirectional associations were examined between daily assessments of rSST variables (total, daytime, evening) with daily assessments of 24-hour behaviors (other-SED, STAND, LPA, MVPA) and with subsequent and previous night SLEEP using a multilevel model (MLM) approach.

**Figure 1:**
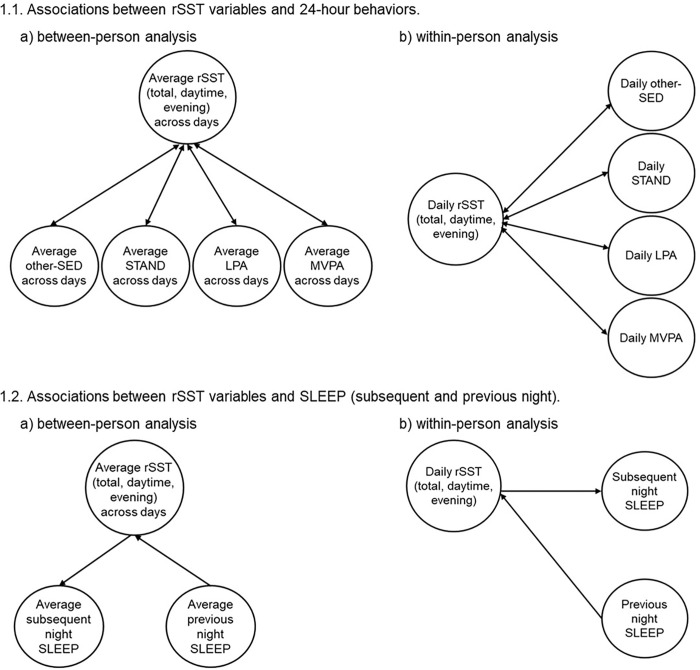
Multilevel models used to examine bidirectional associations between rSST and 24-hour behaviors.

The MLM approach can simultaneously examine the associations of rSST variables and 24-hour behaviors at both between- (level 2: across persons) and within- (across days) person levels. Between-person effects examine how person-level associations exist between 24-hour behaviors (e.g., how an individual’s average total rSST is associated with average levels of 24-hour behaviors). Within-person effects examine how daily associations exist between 24-hour behaviors (e.g., how an individual’s daily departure from their typical total rSST is associated with daily 24-hour behaviors), while controlling for the between-person associations. Separate models were examined for total, daytime, and evening rSST with each 24-hour behavior, other-SED, STAND, LPA, MVPA, and SLEEP (subsequent and previous night). See Fig. 1 for a visual depiction of the models.

Demographic characteristics were included as covariates in the models to control for the individual mean differences when assessing the dependent variables. Age (23–44 years vs. 45–64 years), BMI (normal (≥ 18.5 to < 25) vs. overweight (≥ 25 to < 30) or obese (≥ 30)), sex (female vs. male), chronotype (intermediate vs. morning and evening), day of the week (weekday vs. weekend day), and education (< bachelor’s degree vs. ≥bachelor’s degree) were treated as categorical variables.

The statistical models were conducted in the following two steps, each building upon the previous. Step 1 assessed the main effects associations between clinical and demographic covariates with rSST (total, daytime, evening), other-SED, STAND, LPA, MVPA, and SLEEP. The covariates include age, BMI, sex, chronotype, day of week, and education. Step 1 also covaries the fixed and random effects of intercept, so subsequent models control for individual trajectories of change in the dependent variable.

Step 2 tested the bidirectional associations of rSST variables (total, daytime, evening) with 24-hour behaviors. rSST variables were re-partitioned into between-person and within-person components by calculating person-level means (rSST variable [between-person]) and daily deviations from the person-level mean (rSST variable [within-person]), and then both parameters were simultaneously entered into the model. Variables were centered at the person-mean to address within-person variability. The same approach and models were applied to test the associations of 24-hour behaviors with rSST variables. See Figs. 1 and 2 for a visual overview of the analysis models. For example, the beta coefficients, associated standard errors, and p-values can be interpreted as follows: (a) for total rSST (between-person) - the association between average total rSST with the 24-hour behavior of interest; and (b) for total rSST (within-person) - the daily association between the deviation from the individual’s typical total rSST with the 24-hour behavior of interest (dependent variable).

Additional analyses were also conducted that examined the bidirectional interaction associations between rSST variables (total rSST, daytime rSST, evening rSST) and 24-hour behaviors by the moderators of interest (age, sex, chronotype, and weekend/weekday) that are associated with daily behaviors. The beta coefficients, associated standard errors, and p-values of the interaction effects are interpreted as the level by which the strength of the association of the 24-hour behavior and the dependent variable varies by levels of the putative moderator.

All results from Step 2 and the additional moderator analysis were scaled hourly to aid interpretability. Statistical significance was set at a *p*-value < 0.05. SAS Enterprise Guide, version 8.3, software (SAS Institute, Inc., Cary, North Carolina) was used for analyses.

## RESULTS

The StandUPTV study consented 177 participants, of whom 131 completed baseline measures, and 110 were randomized. Only the 110 randomized participants were considered for this secondary analysis, and the final analytic sample included data from 94 participants who had valid measures based on ≥4 days of ≥10 hours of valid waking wear time from the activPAL and rSST data, and ≥1 valid nightly sleep data. Table 1 presents participants’ clinical and demographic characteristics. The sample had a mean age of42.3±11.5 years, BMI: 29.8±7.8 kg/m^2^, and were 81.9% female. The majority of the sample were adults between the ages of 23–44 years (62.8%), non-Hispanic Whites (77.7%), with an intermediate chronotype (50%), and who earned a bachelor’s degree or higher (76.6%). The means and standard deviation for 24-hour behaviors are presented in Table 2. Additional participant characteristics are provided in Supplemental Table 1 (see Additional File 1), and the main effects of clinical and demographic covariates with 24-hour behaviors are in Supplemental Table 2 (Additional File 1).

### TOTAL rSST AND 24-HOUR BEHAVIORS

#### Associations Between Total rSST with 24-Hour Behaviors

The associations revealed that at the between-person level, each one-hour increase in average total rSST significantly displaced other-SED by 45 minutes and STAND by 10 minutes but was not significantly associated with LPA and MVPA (Table 3). The within-person level associations indicated that each one-hour increase of an individual’s average total rSST significantly displaced other-SED by 43 minutes, STAND by 9 minutes, LPA by 2 minutes, and MVPA by 1 minute.

#### Associations Between Total rSST with Subsequent Night SLEEP

The results indicate that each one-hour increase in average total rSST displaced the subsequent night’s sleep by 3 minutes at the between-person level (Table 5). At the within-person level, each one-hour increase in an individual’s average total rSST increased the subsequent night’s SLEEP by 1 minute. However, the results were not statistically significant.

#### Associations Between 24-Hour Behaviors with Total rSST

The analysis revealed that at the between-person level, each one-hour increase in average other-SED and STAND significantly displaced total rSST by 44 and 34 minutes, respectively, but was not significantly associated with LPA and MVPA (Table 3). The within-person level associations indicated that each one-hour increase of an individual’s average other-SED, STAND, LPA, and MVPA significantly displaced total rSST by 36, 24, 36, and 50 minutes, respectively.

#### Associations Between Previous Night SLEEP with Next Day Total rSST

The results show that each one-hour increase in the previous night’s SLEEP wasn’t significantly associated with the next day’s rSST at the between- or within-person level. (Table 5).

### DAYTIME rSST AND 24-HOUR BEHAVIORS

The analysis between daytime rSST with 24-hour behaviors, at the between-person level, shows that each one-hour increase in average daytime rSST significantly displaced other-SED by 66 minutes and STAND by 15 minutes (Table 4). At the within-person level, each one-hour increase of an individual’s average daytime rSST significantly displaced other-SED by 56 minutes, STAND by 12 minutes, LPA by 3 minutes, and MVPA by 1 minute.

The analysis between 24-hour behaviors with daytime rSST revealed that at the between-person level, each one-hour increase in average other-SED and STAND significantly displaced daytime rSST by 25 minutes and 21 minutes, respectively (Table 4). Associations with LPA and MVPA were not significant. The within-person level associations indicated that each one-hour increase of an individual’s average other-SED, STAND, LPA, and MVPA significantly displaced daytime rSST by 21, 14, 21, and 41 minutes, respectively. Daytime rSST was not significantly associated with the previous or subsequent night’s SLEEP (Table 5).

### EVENING rSST AND 24-HOUR BEHAVIORS

The associations between evening rSST with 24-hour behaviors revealed that at the between-person level, each one-hour increase in average evening rSST significantly displaced other-SED by 85 minutes and STAND by 16 minutes (Table 4). The within-person level associations indicated that each one-hour increase of an individual’s average evening rSST significantly displaced other-SED by 55 minutes, STAND by 12 minutes, and LPA by 3 minutes.

The analysis between 24-hour behaviors with evening rSST revealed that at the between-person level, each one-hour increase in average other-SED and STAND significantly displaced evening rSST by 18 minutes and 13 minutes, respectively (Table 4). The within-person level associations indicated that each one-hour increase of an individual’s average other-SED, STAND, and LPA significantly displaced evening rSST by 15, 10, and 15 minutes, respectively. Increased evening rSST was not associated with the subsequent night’s sleep or the next day’s SLEEP (Table 5).

### INTERACTION ASSOCIATIONS BETWEEN RSST VARIABLES AND 24-HOUR BEHAVIORS

The interaction results between rSST variables and 24-hour behaviors by moderators (age, sex, chronotype, and weekend/weekday) can be found in Supplemental Tables 3–6 (see Additional File 1). For interaction associations between total rSST with 24-hour behaviors, significant negative associations were observed between total rSST with LPA by age (between-person) and by morning chronotype (within-person). Considering daytime rSST and 24-hour behaviors, the interaction associations showed significant negative associations between daytime rSST with other-SED by evening chronotype (within-person), and with LPA by age (between-person), morning chronotype (within-person), and weekend day (within-person). The interaction associations between evening rSST with 24-hour behaviors resulted in significant positive associations between evening rSST with LPA by sex (within-person) and with subsequent night SLEEP by sex (within-person). Results for interaction associations between 24-hour behaviors with rSST variables can also be found in the Supplemental Tables 3–6 (Additional File 1).

## DISCUSSION

This study examined the bidirectional associations between rSST (total, daytime, and evening) and 24-hour behaviors (i.e., other-SED, STAND, LPA, MVPA, SLEEP) using a continuous device-based measures. Overall, greater total rSST was significantly associated with less other-SED and had statistically significant but small associations with STAND, LPA, and MVPA. Negative associations were also observed between other-SED, STAND, LPA, and MVPA with total rSST. Results for daytime and evening rSST and 24-hour behaviors had similar associations as total rSST. No significant associations were observed between rSST variables and SLEEP.

Our results are comparable with previous studies reporting negative associations between rSST with physical activity, resulting in very modest relationships with MVPA.^55,56^ Prior studies have used a combination of self-report, mobile applications, and wearable devices to measure rSST, and have reported overall positive associations between rSST and other-SED.^55–58^ However, the results from this study revealed bidirectional negative associations between rSST variables and other-SED at both between- and within-person levels. Suggesting that increases in rSST variables are associated with lower other-SED and vice versa. For total rSST, the between- (b=−45.7 minutes) and within-person (b=−43.3 minutes) level associations with other-SED are comparable. Whereas, compared to total rSST, the associations between daytime rSST with other-SED are larger at the between- and within-person levels. However, the associations between evening rSST with other-SED are larger compared to both total and daytime rSST at the between- and within-person levels. This suggests, for example, that every hour of evening rSST is associated with the displacement of other-SED by 85 minutes. These findings depict a negative relationship between rSST and other-SED, which contrasts with the current literature suggesting a positive relationship, and this may be due to improved measurement approaches and the consideration of the 24-hour day. Most research studies^56,59,60^ have relied on self-report measures of rSST, with few studies including mobile applications to capture smartphone use^61–64^ or television sounds^58^ as proxies to assess screen time. Similar approaches, with the addition of wearable devices, have also been used to measure other sedentary time, physical activity, and sleep.^55,65,66^ However, several studies assessed smartphone use or TV time, and the combination, along with other screen time contexts, were not included.^61–64^

Rouse and Biddle^67^ assessed specific sedentary behavior contexts among university students and found a negative association between technology (TV, video games, computer)- and study-based sedentary time. Whereas Wagnild and Pollard^59^ compared the activPAL-measured sedentary time and TV time in a sample of pregnant women and found no significant associations between TV time and total sedentary time. However, they reported significant associations between women with high TV time (vs. low TV time) and high evening sedentary time during evening hours (defined as 6pm-11pm).^59^ Several research studies^57,59,60,68^ have suggested that greater rSST contributes to accumulating more overall sedentary time, which research has demonstrated can lead to poor health outcomes. However, the results from this study highlight a different relationship, suggesting that not only could rSST be displacing more active behaviors, but also displacing other types of sedentary time such as reading, spending time with family, friends, etc.

The literature suggests a bidirectional relationship between sleep and rSST, where adults with an evening chronotype, short sleep duration, and insomnia symptoms had higher odds of having high discretionary screen time at follow-up, and vice-versa.^69^ Other research studies have posited a negative association between rSST and sleep duration. Sampasa-Kanyinga et al.^69^ found that evening chronotype, short sleep duration, and insomnia symptoms were associated with more discretionary screen time large population-based prospective cohort. Other research studies have also reported a relationship between more discretionary sedentary screen time and short sleep duration, which we did not find in the present study.^63,70–72^ We only enrolled a sample who were motivated to reduce rSST, and there may not have been enough variance in rSST, and more work is needed to better understand these relationships.

The notable strengths of this study are the novel use of continuous, device-based measures to assess rSST and 24-hour behaviors across consecutive days, and the inclusion of specific behavior contexts of total, daytime, and evening rSST. This work provides a new perspective on the associations between rSST with other-SED and other 24-hour behaviors. The implications of this work provide a better understanding of the relationships between device-based measures of rSST and 24-hour behaviors in sedentary adults, which can support future intervention development in designing optimized interventions addressing multiple health behaviors.

However, this work is not without limitations. The sample was a predominantly non-Hispanic White, highly educated, female population that was inactive and reported high screen time. Participants were encouraged to use the tablet for all recreational screen time, but we could not directly assess smartphone screen time. Beyond rSST, device-based assessment of other sedentary contexts was not measured. Assessing the relationship between long and short bouts of rSST and other-SED would provide further insight into these contexts of sedentary behavior and their relationship with other 24-hour behaviors. Overall, this work provides a new perspective on the associations between rSST and 24-hour behaviors and highlights the need for further work.

## CONCLUSIONS

These results provide a comprehensive perspective between rSST and 24-hour behaviors and present evidence of the bidirectional relationship between rSST and other-SED, and a modest relationship with physical activity. This work used device-based measures to assess continuous rSST and 24-hour behaviors data across consecutive days. Negative associations were observed between rSST variables and other-SED, suggesting that rSST may displace sedentary time rather than contribute to additional total sedentary time. The potential implications of this work suggest that total and specific contexts of sedentary behavior (i.e., total sedentary time and rSST) should be considered individually as behavioral targets in intervention development moving forward. Future interventions that aim to target rSST reduction to increase physical activity should also include strategies that are designed to reduce total sedentary time. More work is needed using device-based measures to understand the influence of rSST on 24-hour behaviors to further understand these behavioral relationships across the 24-hour day and their impact on health outcomes and the potential to inform future public health recommendations for behavior change.

## Supplementary Material

This is a list of supplementary fi les associated with this preprint. Click to download.

• Tables15.docx

• AdditionalFile1.SupplementalTables16.docx

• AdditionalFile2.STROBEstatementchecklistforcrosssectionalstudies.docx

## Data Availability

The datasets used for the current study are available from the corresponding author upon reasonable request.

## Abbreviations

rSST: recreational sedentary screen time
other-SED: other non-rSST sedentary time
STAND: standing
LPA: light-intensity physical activity
MVPA: moderate-to-vigorous physical activity
SLEEP: sleep duration
TV: television viewing
REDCap: research electronic data capture
MEQ: Morningness-Eveningness Questionnaire
E-type: evening chronotype
D-type: intermediate (or day) chronotype
M-type: morning chronotype
kg/m^2^: kilograms/meters^2^ (BMI unit)
WiFi: Wireless fidelity
Hz: Hertz
GGIR: Generation Generic Interface for Research; open-source R-package for sleep data processing
Mg: milligravitational units
HDCZA: the algorithm used in GGIR when the sleep log is not present
MLM: multilevel modeling
SAS: statistical analysis system program
